# Based on bioinformatics analysis lncrna SNHG5 modulates the function of vascular smooth muscle cells through mir‐205‐5p/SMAD4 in abdominal aortic aneurysm

**DOI:** 10.1002/iid3.478

**Published:** 2021-06-29

**Authors:** Han Nie, Wenpeng Zhao, Shizhi Wang, Weimin Zhou

**Affiliations:** ^1^ Department of Vascular Surgery The Second Affiliated Hospital of Nanchang University Nanchang Jiangxi China

**Keywords:** abdominal aortic aneurysm, bioinformatics, ceRNA, lncRNA, vascular

## Abstract

**Objective:**

The aim of this study was to explore expression profiles of long noncoding RNA (lncRNA)‐messenger RNA (mRNA) in abdominal aortic aneurysm (AAA) patients. Further, we explored the mechanisms by which lncRNA SNHG5 modulates the function of vascular smooth muscle cells (VSMC) in AAA.

**Methods:**

Human gene expression profile GSE57691 dataset, was retrieved from Gene Expression Omnibus database. The dataset included gene expression array data of 49 AAA patients and 10 control aortic specimens from organ donors. To explore the main roles of the biological network, differentially expressed lncRNA and mRNAs in the aortic aneurysm (AAA) and normal aortic specimens were determined. Differentially expressed lncRNA and mRNAs were then used to construct a competing endogenous RNA (ceRNA) network using Cytoscape software, and the five key lncRNA were identified. SNHG5 which was significantly downregulated in the AAA was chosen and analysis showed that it regulates mir‐205‐5p and SMAD4 by binding to mir‐205‐5p. Double luciferase reporter gene assays, RNA immunoprecipitation, and RNA knockdown studies were used to establish the relationship between SNHG5 and mir‐205‐5p. Apoptosis rate was determined using flow cytometry, whereas cell proliferation was evaluated using Edu, and 24 well Transwell assay. Western blot analysis was used to determine protein expression levels.

**Results:**

The five differentially expressed lncRNAs were significantly correlated with 34 microRNAs and 112 mRNAs. mRNAs in the ceRNA network are implicated in protein binding, signal transduction, DNA and RNA transcription, development, and cell differentiation. SNHG5 was downregulated in the AAA and acts as a molecular sponge for mir‐205. Downregulation of SNHG5 induces expression of mir‐205‐5p. Increased mir‐205‐5p expression level inhibits SMAD4 production, thus inhibiting proliferation and migration and promotes apoptosis of smooth muscle cells.

**Conclusion:**

Bioinformatics were used to explore molecular mechanism of AAA progression. The findings of this study show that lncRNA SNHG5 regulates proliferation and apoptosis of VSMC cells through modulation of the mir‐205‐5p/SMAD4 axis. Therefore, SNHG5 is a potential therapeutic target for AAA disease.

## Graphical abstract

The findings of this study show that downregulation of SNHG5 increases expression levels of mir‐205‐5p and inhibits SMAD4 expression, thus affecting the function of vascular smooth muscle in abdominal aortic aneurysm. Therefore, SNHG5 is an important factor in pathogenesis of abdominal aortic aneurysm and thus is potential therapeutic target for abdominal aortic aneurysm.

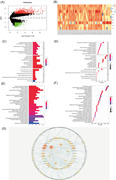

## 1 INTRODUCTION

Abdominal aortic aneurysm (AAA) is an aortic disease with severe effects. Irreversible abdominal aorta radial dilatation caused by various factors is often more than 3 cm or 1.5 times larger compared with the normal diameter.^1^, ^2^ Occurrence and development of AAA are complex processes involving multiple factors and are associated with atherosclerosis, hypertension, chronic obstructive pulmonary disease (COPD), and various proteases. Most AAA patients are asymptomatic and are only diagnosed after the aorta ruptures. Tumor aneurysm rupture is the main cause of high AAA mortality.^3^ Studies report that the overall mortality of ruptured RAAA is approximately 80%.^4^ Occurrence of AAAs is correlate with changes in aortic wall connective tissue. Elastic fibers and collagen mainly determine the mechanical properties of the aorta. In addition, proteoglycans are associated with function of aortic wall tissue.^5^, ^6^, ^7^ However, some previous studies report that collagen degradation is the major cause of aortic rupture.^8^ AAA pathophysiological process is characterized by inflammatory cell infiltration,^9^, ^10^ elastic and collagen fibers degradation,^11^, ^12^ smooth muscle cell death,^13^ arterial wall defects, and increased level of oxidative stress.^1^ However, molecular mechanisms implicated in occurrence and development of AAA has not been fully explored. Therefore, studies should explore the etiology mechanism of AAA generation and development are key in identifying new targets for diagnosis and prognosis of AAA patients thus preventing and improving treatment of AAA.

Long noncoding RNA (ncRNAs) (lncRNA) are noncoding RNAs comprising more than 200 nucleotides.^14^, ^15^ Previous studies report that lncRNAs play an important role in occurrence and development of AAA.^16^, ^17^ For instance, lncRNA SNHG16 inhibits expression of STAT3 by binding to mir‐106b‐5p, thus promoting proliferation of vascular smooth muscle cells (VSMC) and inhibiting apoptosis, ultimately promoting pathogenesis of AAA.^18^ Furthermore, Linc00473 promotes pathogenesis of aneurysm by inhibiting VSMC proliferation and promoting apoptosis through the mir‐212‐5p/BASP1 axis.^19^ However, previous studies have not fully explored the mechanisms lncRNAs promoting pathogenesis of AAA. In this study, data were retrieved from Gene Expression Omnibus (GEO) (GSE57691)^20^ database, and the expression levels of messenger RNA (mRNA), microRNA (miRNA), and lncRNA in AAA were analyzed. Differentially expressed lncRNA were then used to construct a competing endogenous RNA (ceRNA) network. Further, Genetic Ontology (GO) enrichment analysis and Kyoto Encyclopedia of Genes and Genomes (KEGG) pathway analysis was used for functional analysis of differentially expressed lncRNAs implicated in AAA. The findings of this study show that the differentially expressed are lncRNAs are implicated in pathogenesis of AAA. Therefore, these lncRNA can be used as biomarkers for early diagnosis and clinical treatment of AAA.

## 2 MATERIALS AND METHODS

### 2.1 Screening for differential genes

Gene expression profile datasets GSE57691 were obtained from National Center for Biotechnology Information GEO database. Retrieved data included 10 normal aortic tissues and 49 AAAs tissues from GSE57691^20^ human AAA and aortic occlusive disease datasets. Expression levels of genes in the normal group were compared with the AAA group to identify differentially expressed genes (DEGs) using the limma package^21^ in R software. The gene was considered as differentially expressed when the change factor was more than one times (|Fold Change| ≥1) with corrected *p* value (false discovery rate) ≤0.05.

### 2.2 GO and KEGG enrichment analysis

GO and KEGG enrichment analyses were performed using the R language cluster Profiler package.^22^ For pathway analysis, KEGG databases were searched, and Fisher's exact test was used to analyze and determine the significant levels of DEGs enriched in each signaling pathway. Signal pathways significantly associated with DEGs were then identified (*α* = .05).

### 2.3 Construction of lncRNA‐miRNA‐mRNA coexpression network

ncRNAs differentially expressed between normal aortic and AAA tissues were identified. The lncRNA‐miRNA pairs were obtained by comparing them with the StarBase dataset. StarBase^23^ and Target scan database (http://www.targetscan.org) were used to identify miRNA‐mRNA pairs.^24^ The data obtained was then used to construct a lncRNA‐mRNA coexpression network.

### 2.4 Tissue specimen collection

Eight AAA patients (6 males and 2 females with a mean age of 62.3 ± 11.5 years) who underwent surgical resection between 2019 and 2020 were included in this study. Two independent pathologists confirmed histopathological diagnosis for aortic aneurysm based on World Health Organization standards. Fresh tissue samples were obtained and immediately frozen in liquid nitrogen and stored at −80°C for use in RNA extraction. This study was approved by the ethics committee of the Second Affiliated Hospital of Nanchang following the principles of the Helsinki Declaration. Written informed consents were obtained from each participant at the beginning of the study.

### 2.5 Cell line

Human vascular smooth muscle cells (T/G HA‐VSMC) and human renal epithelial cells (HEK‐293T) were obtained from Procell company. 10% fetal bovine serum (GIBCO BRL) was added to DMEM (GIBCO, USA), and all cells were incubated under 5% CO_2_ at 37℃ in two humidified incubators.

### 2.6 Plasmid transfection

Small interfering RNA (siRNA) of SNHG5 (si‐SNHG5), siRNA of NC, mir‐205‐5p mimics and NC were designed by RiboBio. si‐SNHG5 and mir‐205‐5p mimics were cloned into the promoter CMV (Cytomegalovirus) expression vector (Invitrogen) following the manufacturer's instructions, and then transferred to large artery smooth muscle cells. All SNHG5 knockout constructs were acquired from RiboBio. SNHG5 overexpression plasmid (pcdna‐SNHG5) was constructed by cloning the full‐length complementary DNA (cDNA) sequence of SNHG5 into pcDNA3.1 vector (Invitrogen). mir‐205‐5p overexpression plasmid (pcdna‐mir‐205‐5p) was obtained by cloning the full‐length cDNA sequence of mir‐205‐5p into pcDNA3.1 vector (Invitrogen). si‐mir‐205‐5p and SMAD4 mimics were cloned into promoter CMV (Cytomegalovirus) expression vector (Invitrogen) and transfected into arterial smooth muscle cells following the manufacturer's instructions.

### 2.7 Real‐time quantitative PCR (qRT‐PCR)

Total RNA was extracted from tissues or cells using Omega total RNA kit I. Extracted RNA was then translated into cDNA using a reverse transcription Kit (Tiangen). SYBR Green PCR Master Mix Kit (Takara) was used to perform qRT‐PCR on thermal cycle CFX6 system (bio RAD). U6 small nuclear RNA was used as endogenous control. Relative gene expression level was calculated using 2^−ΔΔCt^ method. RNA sequences are shown in Table 1.

**Table 1:** Primer sequences and target sequences used in this study

SNHG5 (5′‐>3′)
Forward primer: CGCTTGGTTAAAACCTGACACT
Reverse primer: CCAAGACAATCTGGCCTCTATC
SMAD4
Forward primer: CTCATGTGATCTATGCCCGTC
Reverse primer: AGGTGATACAACTCGTTCGTAGT
miR‐205‐5p
Forward primer: 5′‐CTTGTCCTTCATTCCACCGGA‐3′
Reverse primer: 5′‐TGCCGCCTGAACTTCACTCC‐3′
U6 snRNA
Forward primer: 5ʹ‐CTCGCTTCGGCAGCACA‐3ʹ
Reverse primer: 5ʹ‐AACGCTTCACGAATTTGCGT‐3ʹ
β‐Actin
Forward primer: 5′‐ATCGTGCGTGACATTAAGGAGAAG‐3′
Reverse primer: 5′‐AGGAAGGAAGGCTGGAAGAGTG‐3′

Abbreviation: snRNA, small nuclear RNA.

### 2.8 Flow cytometry

Smooth muscle cells were cultured in a six‐well plate for 48 h. Annexin V FITC apoptosis detection kit I (BD pharmingen) was used to digest cells with trypsin. A total of 1 × 10^6^ cells/ml cells were obtained from each tube after counting, then centrifuged at 4℃ for 10 min at 1000 rpm, and the supernatant discarded. Cells were then resuspended in 200 μl binding buffer, 10 μl annexin V: FITC added and reacted in under dark conditions for 15 min at room temperature. A total of 300 μl binding buffer and 5 μl PI were then added to the mixture. Apoptosis rate was determined using flow cytometry (BD Biosciences).

### 2.9 Transwell analysis

Transfected cells (1 × 10^5^) and serum‐free medium were inoculated in the upper chamber of 8 μm diameter insert (Merck Millipore), and Dulbecco's modified Eagle's medium (DMEM) medium containing 10% fetal bovine serum was added as a chemotactic agent into the lower chamber. The insert was removed and the liquid dried in the upper chamber after 24 h of incubation in a humidified incubator with 5% CO_2_ at 37℃. The insert was then placed into the hole containing formaldehyde and fixed at room temperature for 30 min. Giemsa staining solution was used for staining at room temperature for 15 min and then the sample was washed twice with phosphate buffered saline. Stained cells were observed and counted under optical microscope. Mean number of migrated cells was recorded in four randomly selected fields in each membrane at ×40 magnification.

### 2.10 Western blot analysis

Total cellular protein was extracted 48 h after transfection using protease‐containing RIPA lysis and phosphatase inhibitors. Proteins were then separated by electrophoresis using sodium dodecyl sulfate‐containing polyacrylamide gels and then transferred to polyvinylidene fluoride membranes. The membrane was blocked with 5% skimmed milk and incubated with primary antibodies (SMAD4 and glyceraldehyde 3‐phosphate dehydrogenase; Abcam) and horseradish peroxidase‐conjugated secondary antibody. The blots were visualized using an ECL Western blot kit (Millipore), following the manufacturers' protocol. The image was used for quantitative analysis of western blot bands obtained.

### 2.11 Luciferase assay

A total of 1 × 10^5^ cells/well (293T) were placed in a 24‐well plate at a 60%–80% confluence density 24 h before transfection. Cells were then transfected using DMEM medium.

Wild‐type (WT) and mutant (Mut) constructs of mir‐205 and SMAD4 mimics or NC were inserted into a luciferase reporter plasmid (Biobio Biotech) 48 h after transfection. Luciferase assay was then performed using Dual‐Luciferase Reporter Assay System (Promega) as following the manufacturer's protocol.

### 2.12 RNA immunoprecipitation assay

Magna RNA binding protein immunoprecipitation kit (Millipore) was used for RNA immunoprecipitation assay. T/G HA‐VSMC cells were lysed in 1 ml RIP lysis buffer supplemented with protease and RNase inhibitors. Cell lysates were then incubated with normal mouse immunoglobulin G (Millipore) or human anti‐ago2 antibody (1:50; Millipore) and rotated overnight at 4°C.  Immunoprecipitated RNAs were then extracted with RNeasy MinElute Cleanup Kit (Qiagen) after treatment with proteinase K buffer and reverse‐transcribed to cDNA. RNA expression levels were analyzed using qRT‐PCR.

### 2.13 EdU incorporation assay

EdU incorporation assay kit (RiboBio) was used to determine cell proliferation rates. In summary, treated cells were seeded in 96‐well plates with a density of 1 × 10^4^ cells/well and incubated with 50 μM EdU for 2 h at 37°C. Cells were fixed with 4% paraformaldehyde, and exposed to 100 μl of 1 × Apollo® reaction cocktail and then incubated with 5 μg/ml Hoechst to stain cell nuclei. Images were captured using fluorescence microscope (Nikon). The percentage of EdU‐positive cells represented the proliferation rate.

### 2.14 Statistical analysis

Data are presented as mean ± *SD*. All statistical analyses were performed using analysis analysis of variancefollowed by Tukey's multiple comparison test on GraphPad Prism 8. Differences with *p* value of less than .05 were considered statistically significant.

## 3 RESULTS

### 3.1 Identification of different lncRNAs, mRNAs, and miRNAs in AAA

**Figure 1:**
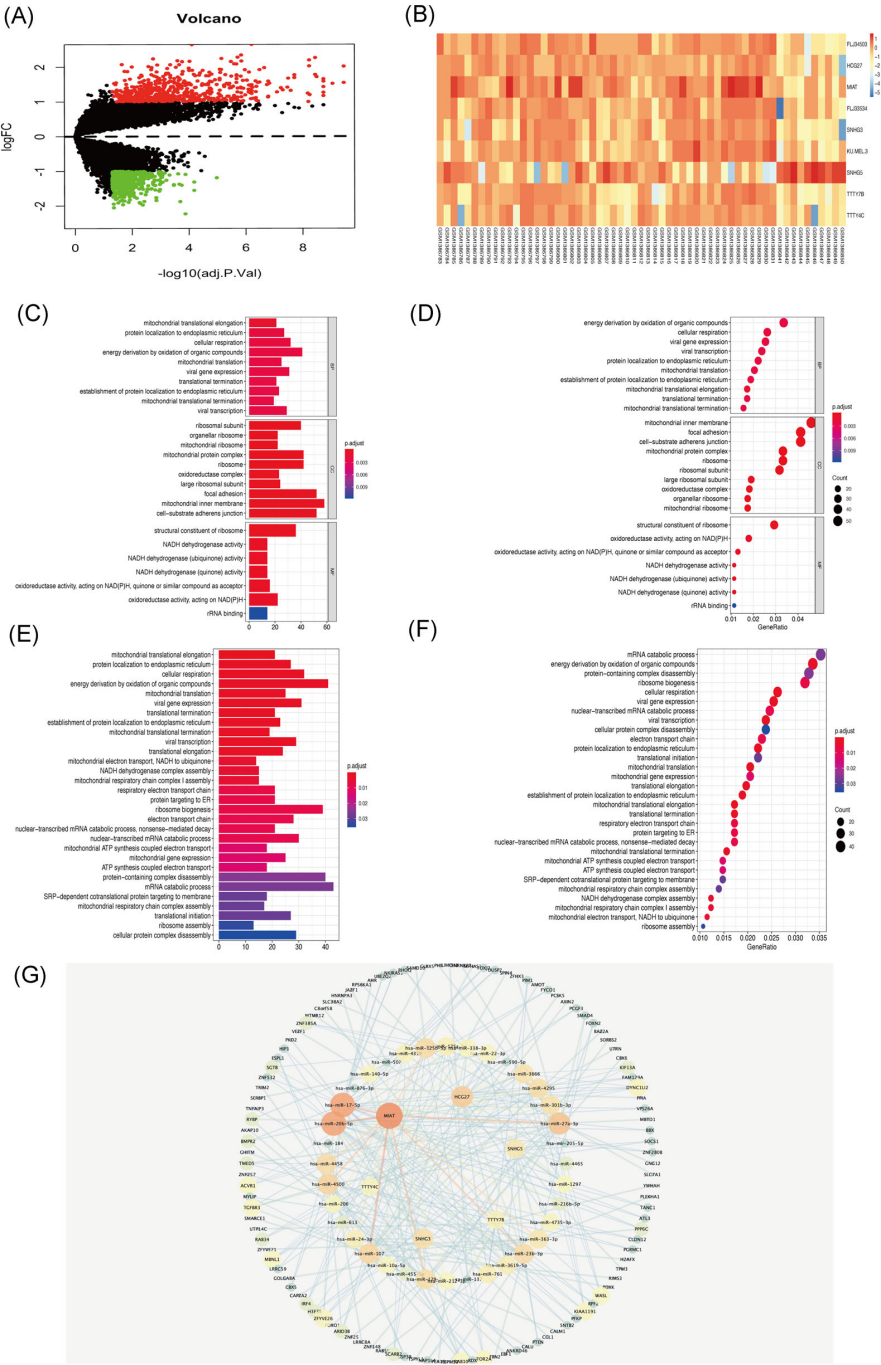
Bioinformatics analysis of abdominal aortic aneurysm. (A) Volcanic map of differentially expressed genes, red represents upregulated genes, green represents downregulated genes, (B) thermogram of nine lncRNAs in the sample, the deeper the color, the higher the expression. (C) GO enrichment analysis histogram of differential mRNA.(D) GO enrichment analysis dot plot of the differentially expressed mRNA. (E) KEGG enrichment analysis histogram of differential mRNA. (F) KEGG enrichment analysis dot chart of differential mRNA. (G) The outer layer is mRNA, the inner layer is miRNA, and the innermost layer is lncRNA. GO, Genetic Ontology; KEGG, Kyoto Encyclopedia of Genes and Genomes; lncRNA, long noncoding RNA; mRNA, messenger RNA; miRNA, microRNA

Limma package standardizes gene expression profile data using the R statistical program. The multiple (|Fold Change|) ≥2 times or ≤−0.5 times change was chosen, and the corrected *p* value was set at ≤.05 mRNA to identify differentially expressed noncoding (nc) RNA. A total of 1330 differential mRNAs and 9 differential lncRNAs were identified in the AAA group compared with normal aortic tissues (Figure 1A,B) (Table 2).

**Table 2:** differential lncRNA in abdominal aortic aneurysm

gene	logFC	AveExpr	*t*	*p* Value	adj.*p* Value	*B*
FLJ34503	1.39983977	−0.1375964	7.79810576	1.05E‐10	5.82E‐08	14.2072475
HCG27	1.59136977	−0.1974604	7.5459844	3.43E‐10	1.62E‐07	13.0494934
MIAT	1.54898333	0.14270018	5.17878285	3.11E‐06	0.00012142	4.41303234
FLJ33534	1.64506519	−0.2433179	4.99919234	6.36E‐06	0.00020782	3.7343407
SNHG3	1.44023055	−0.4041352	4.74414156	1.33E‐05	0.00035945	2.95524029
KU‐MEL‐3	1.13372969	−0.2499761	4.03703643	0.00016251	0.0022036	0.62534546
SNHG5	−1.8721998	−0.4611691	−3.8629573	0.00033329	0.00375801	−0.0325704
TTTY7B	1.04273442	−0.4468529	3.74814298	0.00040599	0.00431825	−0.2655008
TTTY4C	1.27300079	−0.4613613	3.61998698	0.00061098	0.00577308	−0.6167421

Abbreviation: lncRNA, long noncoding RNA.

### 3.2 Functional prediction of differentially expressed mRNA in AAA

GO and KEGG analyses of the 1330 differentially expressed mRNA were performed using David tool. Go analysis showed that the differently expressed mRNAs are mainly involved in apoptosis, transcriptional regulation, protein binding, metal ion binding, enzyme binding, and actin‐binding (Figure 1C,D). KEGG pathway analysis showed that the differentially expressed mRNAs are mainly implicated in pluripotency signaling pathway, Hippo signaling pathway, cancer microRNAs, meiosis, islet signaling pathway and endocytosis (Figure 1E,F).

### 3.3 Construction of AAA lncRNA‐miRNA‐mRNA network

Bioinformatics tools were used to analyze and construct lncRNA dominated ceRNA network to explore the role of lncRNAs regulating miRNA in AAAs. Starbase dataset was used to screen lncRNA miRNA interaction pairs. In addition, mir code (a map of putative microRNA target sites), Starbase (Starbase), and target scan databases (http://www.targetscan.org) were used to identify miRNA mRNA pairs. lncRNA‐miRNA‐mRNA interaction network was constructed using Cytoscape tool. The network comprised 5 lncRNA nodes, 34 miRNA nodes, 112 mRNA nodes, and 275 edges (Figure 1G). The major lncRNA in the ceRNA network (each interacting with more than five distinct miRNAs) were five upregulated lncRNAs including TTTY7B, HCG27, MIAT, TTTY4C, SNHG3, and one downregulated lncRNA, SNHG5.

**Figure 2:**
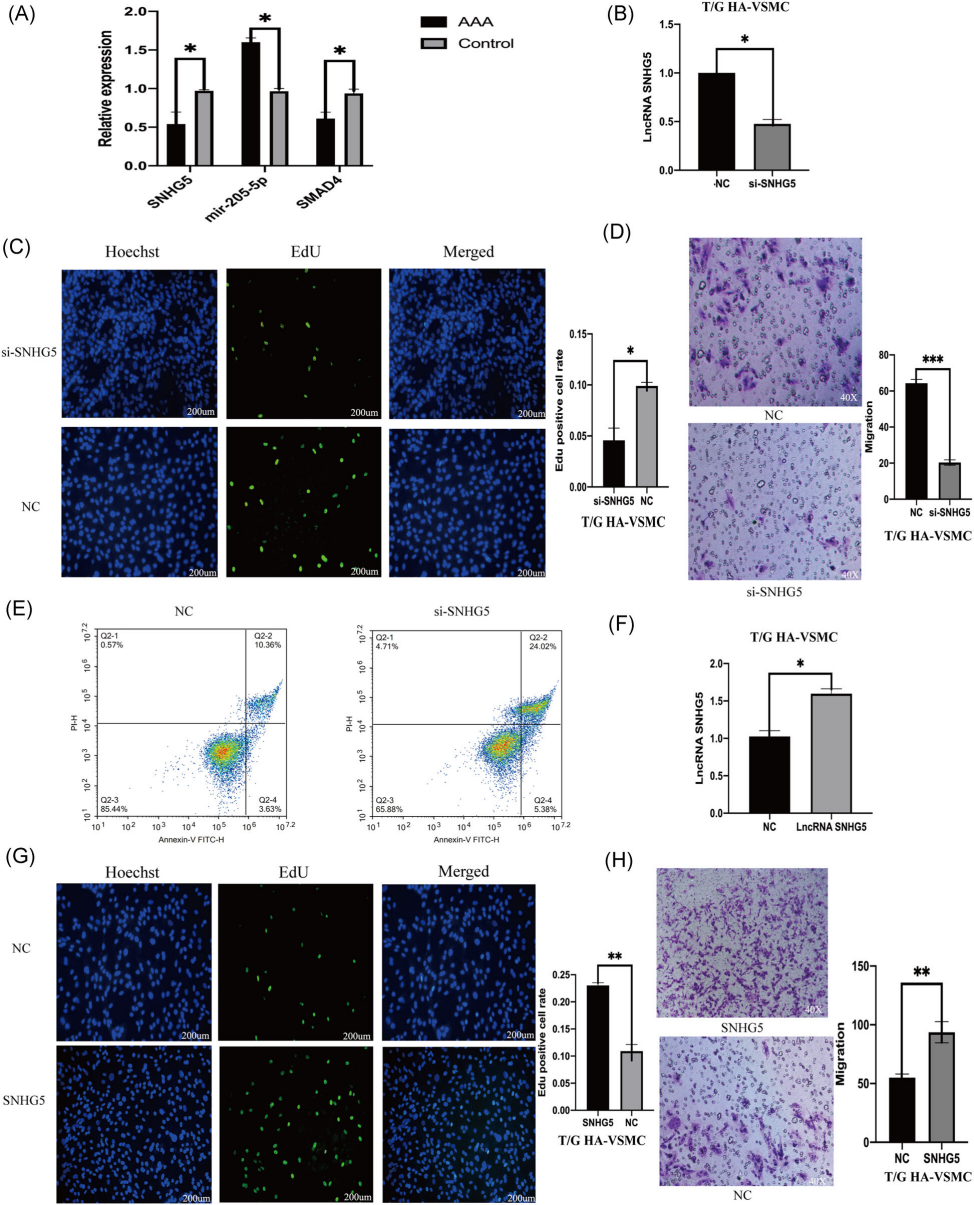
Knockdown of SNHG5 inhibits VSMC proliferation, migration and promotes apoptosis. (A) SNHG5, mir‐205‐5p, and SMAD4 examined using qRT‐PCR in eight AAA pairs and matched with adjacent abdominal aorta tissues, (B) SNHG5 knockdown in T/G HA‐VSMC cells evaluated using qRT‐PCR. (C) the SNHG5 proliferation ability silencing T/G HA‐VSMC evaluated using Edu assay (200 µm). (D) the migration ability of SNHG5 silenced T/G HA‐VSMC was evaluated using Transwell assay. (E) the T/G HA‐VSMC silencing SNHG5 evaluated using flow cytometry. (F) SNHG5 expression in T/G HA‐VSMC cells detected using qRT‐PCR. (G) The proliferation ability of T/G HA‐VSMC overexpressing SNHG5 evaluated using Edu assay (200 µm). (H) The migration ability of T/G HA‐VSMC overexpressing SNHG5 evaluated using Transwell assay. The results are presented as mean ± *SD* for three independent experiments. Each experiment performed in triplicate. *p < .05, **p < .01, ***p < .001. qRT‐PCR, real‐time quantitative PCR

Expression levels of SNHG5, mir‐205‐5p, and SMAD4 in human AAA were determined using qPCR. Analysis of qPCR results showed that expression level of SNHG5 and SMAD4 in AAA was significantly lower, whereas mir‐205‐5p expression level was significantly higher compared with expression levels in normal aorta (Figure 2A).

### 3.4 The decrease in lncRNA SNHG5 expression can inhibit the proliferation and migration of VSMC and promote apoptosis

**Figure 3:**
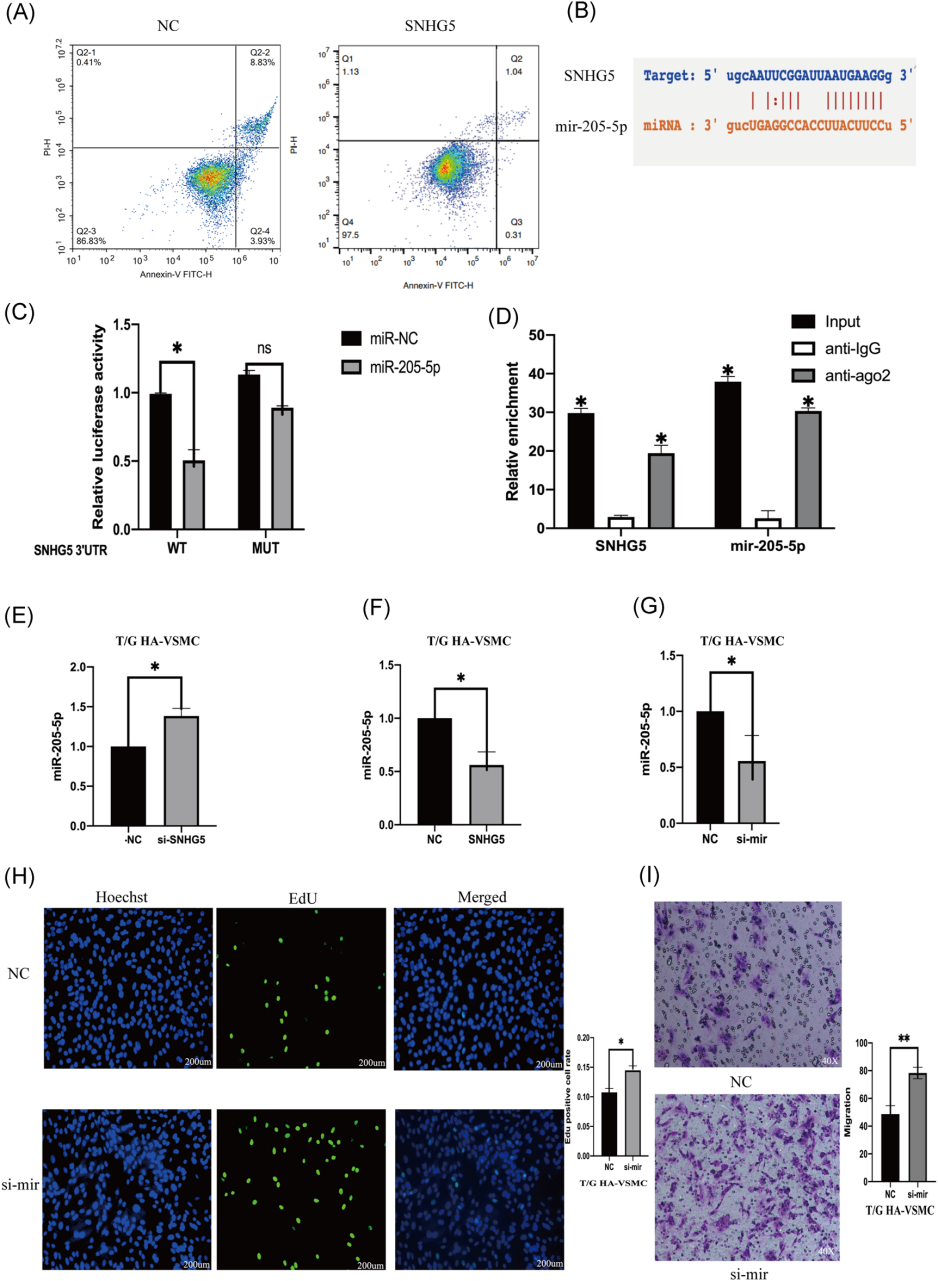
mir‐205‐5p is negatively regulated by lncRNA SNHG5. (A) The apoptosis of T/G HA‐VSMC overexpressing SNHG5 evaluated using flow cytometry. (B) SNHG5 potential binding site with mir‐205‐5p in SNHG5 sequence of lncRNA. (C) binding characteristics between SNHG5 and mir‐205‐5p analyzed using double luciferase report, *p < .05. (D) RNA immunoprecipitation was used as a control to enrich SNHG5 and mir‐205‐5p in anti ago2 group and anti‐IgG. *p < .05. (E) The mir‐205‐5‐p expression of in T/G HA‐VSMC cells after SNHG5 knockdown was examined using qRT‐PCR. (F) mir‐205‐5 p expression after SNHG5 overexpression in T/G HA‐VSMC cells evaluated using qRT‐PCR. (G) the mir‐205‐5p knockdown effect in T/G HA‐VSMC cells was examined using qRT‐PCR. (H) proliferation ability of mir‐205‐5p silenced T/G HA‐VSMC was evaluated using Edu assay(200 µm). (I) mir‐205‐5p silenced by Transwell assay Migration ability of T/G HA‐VSMC. The results are presented as mean ± *SD* for three independent experiments. Each experiment performed in triplicate. *p < .05, **p < .01, ***p < .001. IgG, immunoglobulin G; lncRNA, long noncoding RNA

To explore the role of lncRNA SNHG5 in AAA, lncRNA SNHG5 knockdown was performed in T/G HA‐VSMC cells by siRNA which was then verified by qPCR (Figure 2B). Proliferation and migration of VSMC cells decreased significantly after SNHG5 knockdown (Figure 2C,D). Flow cytometry analysis showed that SNHG5 knockdown affects on proliferation and migration of VSMCS is through promotion of apoptosis (Figure 2E). T/G HA‐VSMC cells were transfected with SNHG5 sequence containing plasmid and qRT‐PCR was then performed to determine the expression level of SNHG5 (Figure 2F). The results showed that overexpression of SNHG5, significantly increases proliferation and migration ability of VSMCs (Figure 2G,H). Flow cytometry analysis showed that effect of overexpression of SNHG5 on proliferation and migration of VSMCS is through inhibition cell of apoptosis (Figure 3A).

### 3.5 lncRNA SNHG5 sponges mir‐205‐5p in vascular smooth muscle cells

**Figure 4:**
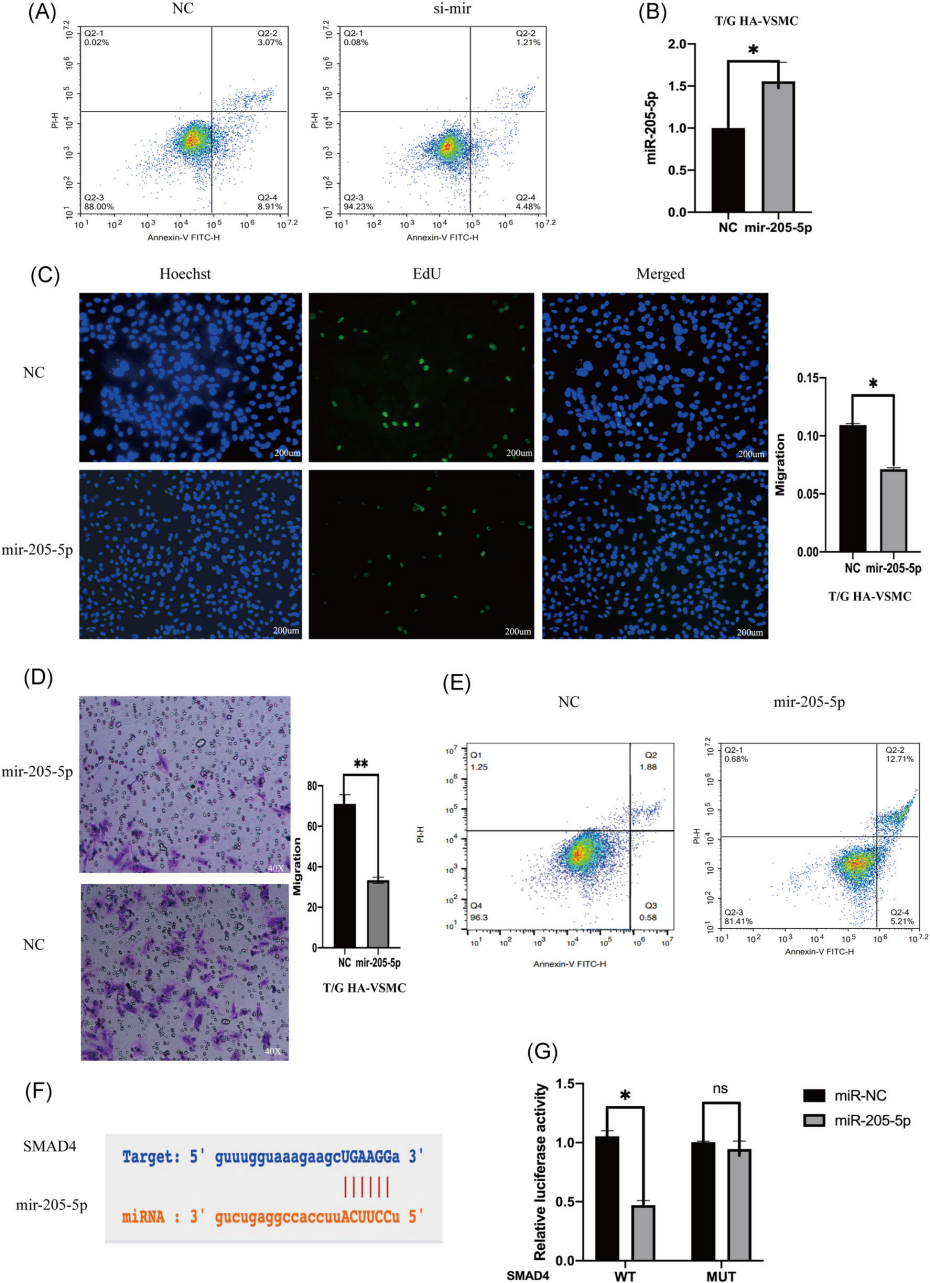
mir‐205‐5p inhibits VSMC proliferation, migration, and promotes apoptosis; SMAD4 is negatively regulated by mir‐205‐5p. (A) The apoptosis of mir‐205‐5p silenced T/G HA‐VSMC cells was evaluated by flow cytometry (FCM). (B) the mir‐205‐5p overexpression effect in T/G HA‐VSMC cells was examined using qRT‐PCR. (C) the mir‐205‐5p proliferation ability overexpressed T/G HA‐VSMC cells evaluated using Edu assay (200 µm). (D) the migration ability of mir‐205‐5p overexpressing T/G HA‐VSMC was evaluated by Transwell assay. (E) The overexpression of mir‐205‐5p was evaluated by flow cytometry. (F) the potential site of SMAD4 targeted binding in mir‐205‐5p sequence. (G) Double Luciferase Report was used to analyze the binding characteristics of SMAD4 and mir‐205‐5p, *p < .05. The results are presented as mean ± *SD* for three independent experiments. Each experiment performed in triplicate. *p < .05, **p < .01, ***p < .001. qRT‐PCR, real‐time quantitative PCR

The role of SNHG5 in complementing activity of mir‐205‐5p was explored based on interactions from the previous ceRNA network (Figure 3B). Double luciferase assay results showed that overexpression of mir‐205‐5p decreases SNHG5‐wt luciferase activity, however, overexpression of mir‐205‐5p had no significant affect on SNHG5‐mt luciferase activity (Figure 3C). Protein immunoprecipitation analysis showed that ago2 antibody can inhibit lncRNA SNHG5 and mir‐205‐5p (Figure 3D), implying that SNHG5 binds to mir‐205‐5p to exert its activity. LncRNA SNHG5 knockdown in VSMCs increased mir‐205‐5p expression level (Figure 3E). On the other hand, SNHG5 overexpression decreased expression of mir‐205‐5p (Figure 3F). Mir‐205‐5p knockdown in T/G HA‐VSMC cells was achieved by inhibitors and expression level mir‐205‐5p was evaluated using qRT‐PCR (Figure 3G). Proliferation and migration abilities of VSMCs were significantly improved after mir‐205‐5p knockdown (Figure 3H,I). Flow cytometry analysis showed that mir‐205‐5p knockdown decreases apoptosis (Figure 4A). T/G HA‐VSMC cells were transfected with a plasmid containing mir‐205‐5p sequence and the expression level of mir‐205‐5p was determined using qRE‐PCR (Figure 4B). Overexpression of mir‐205‐5p significantly reduced proliferation and migration rates of VSMCs (Figure 4C,D). Flow cytometry results showed that effect of overexpression of mir‐205‐5p on proliferation and migration of VSMCS is through promotion of cell apoptosis (Figure 4E).

### 3.6 Downregulated lncRNA SNHG5 inhibits SMAD4 expression by upregulating mir‐205‐5p

**Figure 5:**
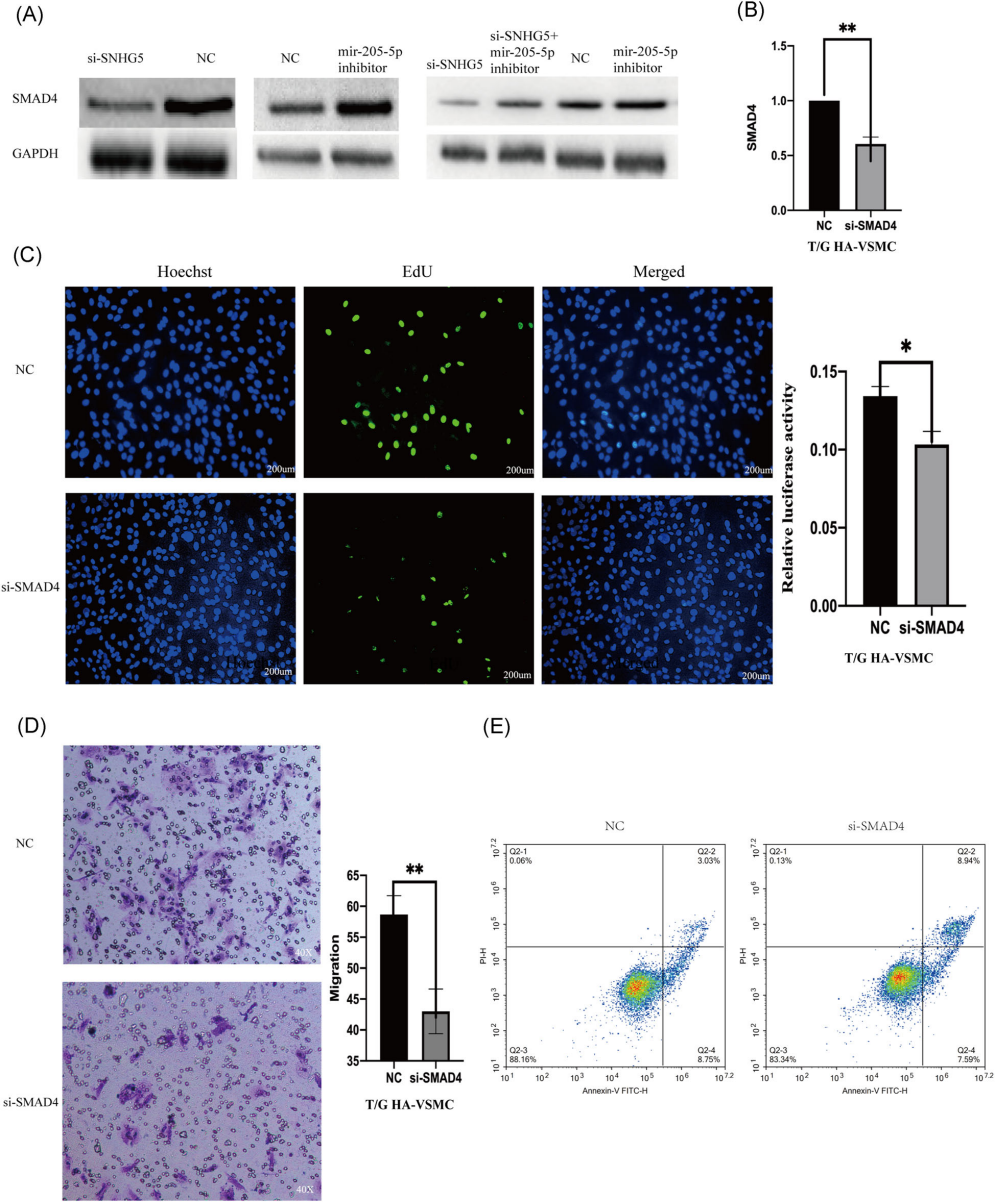
Down regulation of lncRNA SNHG5 alleviates the promotion of SMAD4 by downregulation of mir‐205‐5p expression. (A) Western blot used to analyze the effect of SNHG5 and mir‐205‐5p on the SMAD4 expression. (B) qRT‐PCR used to examine the SMAD4 silencing effect in T/G HA‐VSMC cells. (C) The proliferation ability of SMAD4 silenced T/G HA‐VSMC was evaluated using Edu assay (200 µm). (D) The migration ability of SMAD4 silenced T/G HA‐VSMC was evaluated using Transwell assay. (E) The apoptosis of SMAD4 silenced T/G HA‐VSMC was evaluated using flow cytometry. The results are presented as mean ± *SD* for three independent experiments. Each experiment performed in triplicate *p < .05, **p < .01, ***p < .001. lncRNA, long noncoding RNA

**Figure 6:**
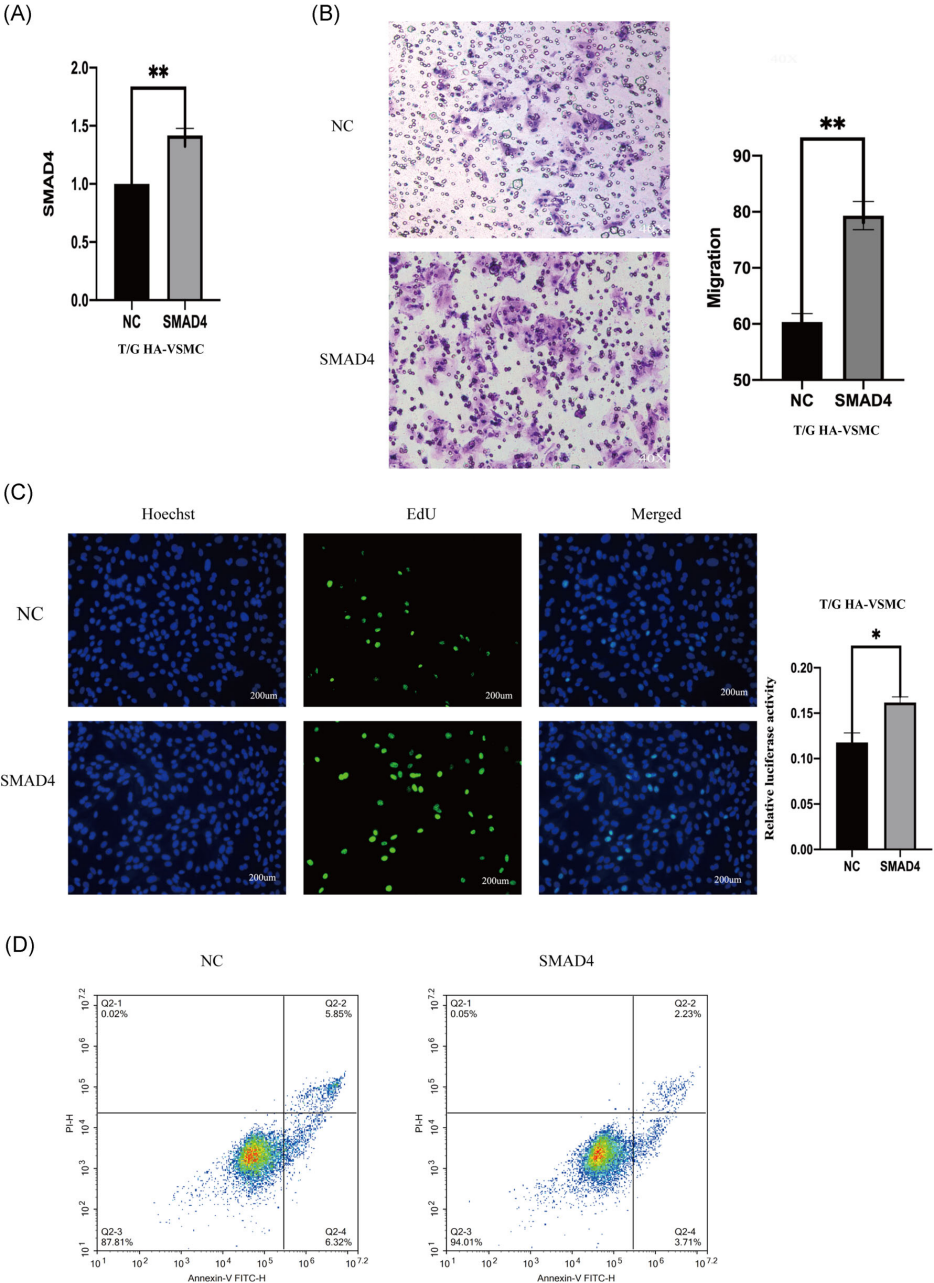
SMAD4 promote VSMC proliferation, migration, and promotes apoptosis. (A) Methods: the effect of SMAD4 overexpression in T/G HA‐VSMC cells was verified by qRT‐PCR. (B) The migration ability of SMAD4 overexpressing T/G HA‐VSMC evaluated using Transwell assay. (C) the proliferation ability of SMAD4 overexpression T/G HA‐VSMC cells was evaluated by Edu assay(200 µm). (D) The withering rate of overexpressing T/G HA‐VSMC SMAD4 was evaluated using flow cytometry. The results are presented as mean ±* SD* for three independent experiments. Each experiment performed in triplicate. *p < .05, **p < .01, ***p < .001. qRT‐PCR, real‐time quantitative PCR

The role of mir‐205‐5p on complementing activity of SMAD4 was explored based on interactions from the previous ceRNA network (Figure 4F). Double luciferase assay results showed that SMAD4 overexpression decreases mir‐205‐5p‐wt luciferase activity but did not significantly affect mir‐205‐5p‐mt luciferase activity (Figure 4G). Moreover, SMAD4 expression level was affected by mir‐205‐5p and lncRNA SNHG5. LncRNA SNHG5 knockdown significantly downregulated SMAD4 expression, whereas mir‐205‐5p inhibition reversed this effect (Figure 5A,B). SMAD4 knockdown significantly decreased migration and proliferation of VSMCs (Figure 5C,D). Flow cytometry showed that SMAD4 knockdown increases apoptosis rate (Figure 5E). On the other hand, overexpression of SMAD4 significantly increased proliferation and migration rates of VSMCs (Figure 6A–C). Flow cytometry results showed that effect of SMAD4 overexpression on proliferation and migration of VSMCS is through inhibition of cell apoptosis (Figure 6D).

## 4 DISCUSSION

AAA is a common degenerative aorta disease, highly prevalent in 65 years people and above.^25^, ^26^ AAA patients are usually asymptomatic until rupture of the aorta occurs, which is mostly fatal.^27^, ^28^ Occurrence and development of AAA are complex processes involving multiple factors and are directly associated with atherosclerosis, hypertension, COPD, and various proteases.^29^ Previous studies report that lncRNA is implicated in several diseases by regulating transcription and stability of target gene mRNAs,^30^, ^31^ such as ANRIL, SENCR, and HIFLA‐ASL.^32^, ^33^, ^34^ lncRNAs affect occurrence and development of AAA by regulating related mRNA. However, role of lncRNA in AAA and its mechanism of action have not been fully explored. Therefore, studies should explore lncRNAs that are differentially expressed in AAA to understand the development of AAA.

In this study, differentially expressed RNAs in AAA were screened using GEO database. AAA ceRNA network was then constructed using the GEO dataset combined with data from public databases. Analysis showed that SNHG5 modulates SMAD4 expression by regulating mir‐205‐5p, thus affecting occurrence and development of AAA. PCR analysis confirmed decrease in expression of SNHG5 and SMAD4, whereas mir‐205‐5p expression was high in AAA group, which is consistent with the ceRNA network results. Further, SNHG5 knockdown increased VSMC apoptosis and inhibited proliferation and migration of VSMC. Previous studies report that SNHG5 binds to mir‐205‐5p. Luciferase results showed that SNHG5 binds to mir‐205‐5p, and inhibition of SNHG5 increases mir‐205‐5p expression. Findings from our previous studies show that mir‐205‐5p is upregulated in AAA. Further, apoptosis of VSMC is increased, and proliferation and migration ability decreases after upregulation of mir‐205‐5p, which is consistent with findings from a previous study by Kim.^35^ Moreover, inhibition of SNHG5 increase expression levels of mir‐205‐5p, promotes apoptosis and inhibits proliferation. These findings indicate that SNHG5 affects vascular smooth muscle function by interacting with mir‐205‐5p. SMAD4 is the only common coding protein in SMAD family mammals that mediate and plays an important role in BMP and transforming growth factor beta (TGF‐β) pathway.^36^ Previous studies report that SMAD4 plays an important role in vascular remodeling, development and maturation of vascular endothelium, and development of the heart.^37^, ^38^, ^39^, ^40^ Notably, SMAD4 affects proliferation and migration of SMC, and SMC lacking SMAD4 recruits macrophages by producing chemokines, leading to aortic inflammation.^41^ SMAD4 knockdown in VSMC significantly increased apoptosis and decreased proliferation and migration capacity of these cells. These findings show that SMAD4 binds to mir‐205‐5p and increases expression of SMAD4. In addition, SNHG5 downregulation reduces the effect of mir‐205‐5p on SMAD4 expression, significantly increasing apoptosis of VSMC, and reducing proliferation and migration of VSMC.

Vascular smooth muscle cells are the main components of the aortic wall. Therefore, changes in functions of VSMCs significantly affects the stability of the aortic wall. Previous studies report that VSMC can synthesize and secrete lysyl oxidase, which is implicated in formation of fibrous structure, and participates in formation of MMP and TIMP. The integrity of the extracellular fibrous structure of the aortic wall can thus be controlled through these processes.^42^, ^43^ Previous studies report that regulation of the function of VSMC modulates occurrence and development of AAA. For instance, downregulation of SOX2OT alleviates development of AAA by inhibiting apoptosis of VSMC.^44^ In addition, SENCR inhibits development of AAA by inhibiting apoptosis of VSMC and degradation of extracellular matrix.^45^ Further, mir‐26 can promotes proliferation of VSMC, inhibits apoptosis and modulates TGF‐β pathway to promote development of AAA.^46^ The findings of this study show that downregulation of SNHG5 inhibits expression of SMAD4 through mir‐205‐5p, thus significantly increasing apoptosis of VSMC, and reducing proliferation and migration rates of VSMC. These findings imply that lncRNA SNHG5/mir‐205‐5p/SMAD4 participate in occurrence and development of AAA by modulating the function of VSMC. Therefore, SNHG5/mir‐205‐5p/SMAD4 are attractive biomarkers for the development of novel therapies for AAA.

## 5 CONCLUSION

The findings of this study show that downregulation of SNHG5 increases expression levels of mir‐205‐5p and inhibits SMAD4 expression, thus affecting the function of vascular smooth muscle in AAA. Therefore, SNHG5 is an important factor in pathogenesis of AAA and thus is potential therapeutic target for abdominal aortic aneurysm.

## CONFLICT OF INTERESTS

The authors declare that there are no conflict of interests.

## AUTHOR CONTRIBUTIONS

**Han Nie:** research design and drafting the manuscript. **Weimin Zhou:** review and revision of the manuscript and writing guidance.

## ETHICAL APPROVAL AND CONSENT TO PARTICIPATE

1The study was approved by the medical ethics committee of the Second Affiliated Hospital of Nanchang University. All patients involved in the study signed the informed consent voluntarily. All methods were performed in accordance with the relevant guidelines and regulations.

## Data Availability

The datasets used and/or analysed during the current study are available from the corresponding author on reasonable request.

## Footnotes

1 The authors’ research was supported by the research fund of The National Natural Science Foundation of China (82060095)
