# Corrigendum

**DOI:** 10.1002/iid3.674

**Published:** 2022-06-30

1

In Nie et al.,^1^ there was an error in Figure 2D on page 1313.

Figure 2

**Figure:**
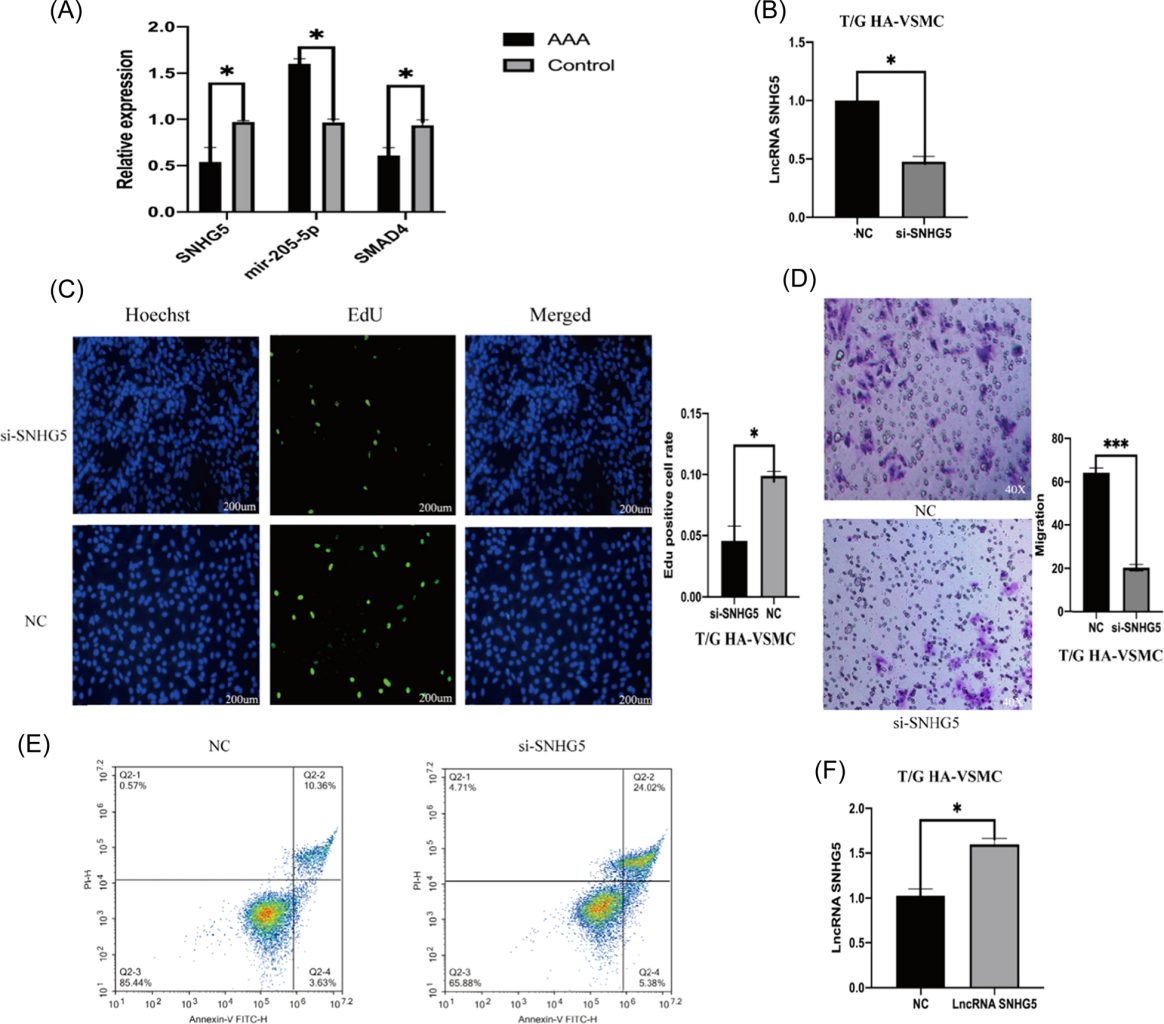

**Figure:**
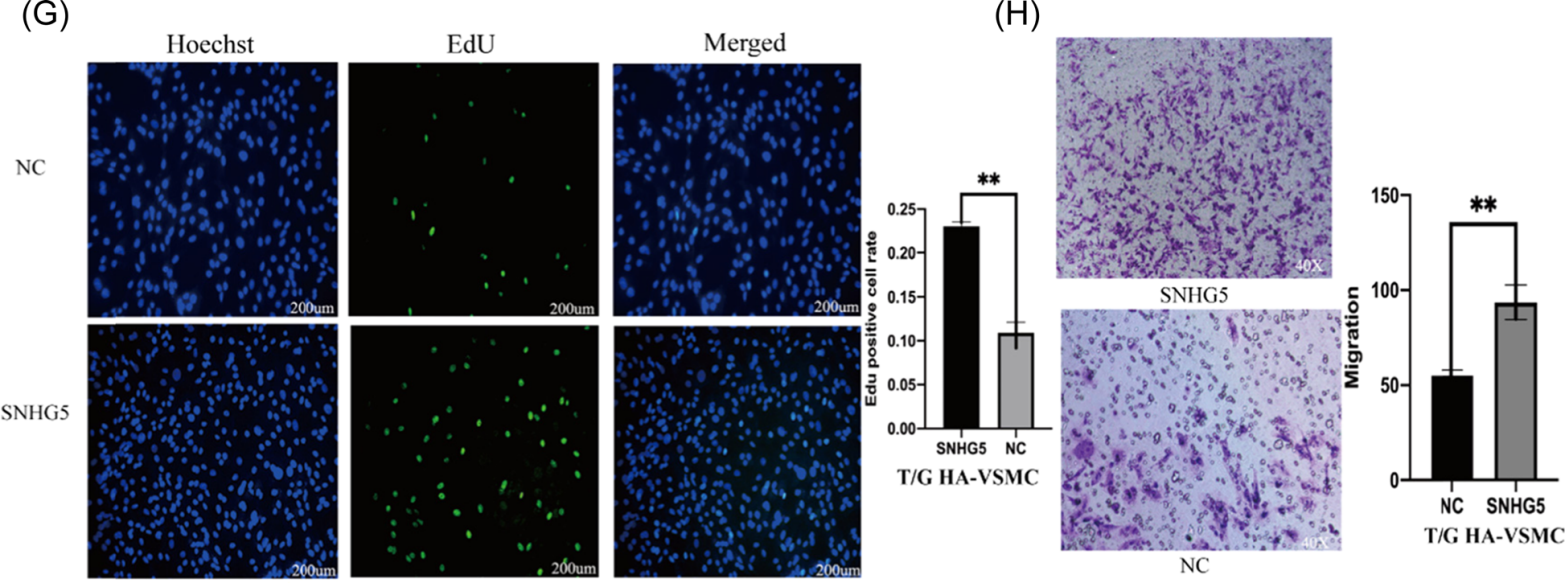

The correct Figure 2 is shown below. The authors confirm that the results and conclusion of the article remain unchanged.

We apologise for this error.
